# Declining trends in smokeless tobacco use among Indian women: findings from global adult tobacco survey I and II

**DOI:** 10.1186/s12889-021-12089-6

**Published:** 2021-11-09

**Authors:** Shishirendu Ghosal, Abhinav Sinha, Srikanta Kanungo, Sanghamitra Pati

**Affiliations:** Division of Public Health, ICMR-Regional Medical Research Centre, Chandrasekharpur, Bhubaneswar, Odisha -751023 India

**Keywords:** GATS, India, Oral Cancer prevention, Smokeless tobacco, Women

## Abstract

**Background:**

Smokeless Tobacco (SLT) use is culturally rooted and more acceptable among women in India. SLT is a significant risk for oral cancers and has other adverse health outcomes on women’s general as well as reproductive health. This study aimed to estimate and compare the prevalence and correlates of SLT among adult females in India using Global Adult Tobacco Survey (GATS), 2009–2010 (GATS 1) and 2016–2017 (GATS 2).

**Methods:**

Data from a nationally representative cross-sectional study GATS 1 (*n* = 35,529) and GATS 2 (*n* = 40,265) were analysed for adult female smokeless tobacco users. Correlates of SLT exposure were assessed separately using binary logistic regression. Multivariable logistic regression analysis was done for the variables which computed *p* < 0.1. The association was expressed as Adjusted Odds ratio with 95% confidence intervals.

**Results:**

There was a reduction in prevalence of SLT use among women in India between GATS 1 (18.4%) and GATS 2 (12.8%). SLT use was highest among the North-Eastern women in both rounds [AOR: 4.567 (3.942–5.292) during GATS-1 and 9.149 (7.722–10.839) during GATS-2]. Odisha had highest prevalence of 56.53% while Himachal Pradesh had lowest 0.14% during the recent GATS 2 survey. 33.3% vs. 34.80% of the participants were willing to quit tobacco in Central region across both rounds of survey.

**Conclusions:**

Although, smokeless tobacco prevalence among females has reduced between 2009 and 2016 in India, yet tobacco control strategies need further pace. Hence, more focused gender-based tobacco control programs and policies are the need of time.

## Background

Smokeless tobacco (SLT) refers to the products which are consumed without combustion through chewing, spitting, dipping, snuffing and applying on teeth and gums [1, 2]. There are 248 million SLT users globally, 90% of whom live in Indian subcontinent [3]. Tobacco consumption is more widespread amongst males but, it is evident that young girls and women tend to use SLT more as compared to smoking [4]. Behavioral science attributes socio-cultural practices and acceptability of SLT as one of the reasons for extensive SLT use amongst females [5, 6].

SLT is a potential threat for women’s general as well as reproductive health [7]. Regular SLT consumption leads to several adverse health outcomes such as oro-pharyngeal cancers, heart diseases, osteoporosis, reproductive morbidities including infertility and pregnancy complications [8, 9]. Its use during pregnancy can lead to pre-term delivery, low birth weight babies, stillbirth and may act as neuro-teratogen affecting development of nervous system in the fetus [10, 11]. In general, SLT use has also been associated with compromised nutritional status leading to weak immunity and increased vulnerability to infections [12, 13].

SLT is one of the leading cause of potentially malignant oral soft tissue lesions such as leukoplakia, erythroplakia and oral submucous fibrosis; eventually leading to oral cancers [14]. Various studies suggest a strong causal relation between SLT use and oral cancer [15]. Oral cancer is one of the top three cancers accounting 30% of all cancers in India with an incidence of 1,19,992 cases and 72,616 deaths every year [16, 17].

Several national level studies like National Family Health Survey (NFHS), National Sample Survey (NSS) and National Household Survey of Drug and Alcohol Abuse in India (NHSDAA) captures information on tobacco use to assist in forming evidence based effective tobacco control policies. The NFHS survey collected tobacco related data since its second round. The fourth round of NFHS survey (2015–2016) estimated the prevalence of tobacco use amongst adult women (*n* = 699,686 in 601,509 households) to be 6.8% (4.4% in urban and 8.1% in rural) [18]. Most of these studies lacked comprehensive data on tobacco use among adults. They either focused on local data with urban bias or were a part of other health-based surveys with less focus on tobacco related behavior. In India, Global Adult Tobacco Survey (GATS) was the first nationally representative survey that captured specific tobacco related information. This country wide survey provides a reliable data on tobacco use among adults aged 15 years and above, its correlates including socio-economic, demographic and other key indicators.

Improved education and decreased cultural restrictions have increased the spending capacity of women who have eventually become a soft target group for tobacco industry [6]. Therefore, it is imperative to understand the burden and patterns of SLT use among women so as to formulate gender-based tobacco control policies and programs. The change in trends and burden of SLT use over the years needs to be monitored, to evaluate our existing policies. Hence, the present study was planned to compare two rounds of GATS India data with the following objectives:

1. To estimate and compare the prevalence of smokeless tobacco use among adult females in India using a nationally representative sample from GATS1, 2009–2010 and GATS2, 2016–2017.
2. To determine and compare the correlates (socio-economic, demographic) of smokeless tobacco use among adult females in India using data from both rounds of GATS survey.

## Methods

### Overview of data

The Global Adult Tobacco Survey (GATS) is the global standard for monitoring adult tobacco use in a systematic way which tracks key indicators of tobacco control. GATS surveys a nationally representative cross-sectional household based adult samples, aged 15 years and more, using a standard core questionnaire, sample design, and data collection and management procedures for each country. In India, GATS 1 surveyed 29 states and two Union Territories while GATS 2 surveyed 30 states and two Union Territories covering 69,296 & 74,037 individuals respectively. Stratified multi-stage cluster sampling design was used to identify the specific households for GATS survey. Each state and within each state, rural and urban areas were selected separately for sampling. Primary sampling Unit (PSU) comprised of wards in urban areas which were selected by a three-stage process. During the first stage, a checklist of all wards of towns and cities of each state were obtained which formed the sampling frame. Wards, required for sample, were selected by probability proportional to size (PPS) sampling method. In the next stage, a list of all census enumeration blocks (CEB) were obtained from which one CEB per selected ward was chosen by PPS. In the third stage, required number of residential households were selected from each CEB. In rural areas, households were selected through a two-stage sampling process where villages were PSU, selected through PPS. In the second stage, required number of households were selected from each village.

Amongst the enrolled households, Household Questionnaire was administered to determine the GATS eligibility requirements and to make a list of all eligible residents of the household. Further, one adult was randomly selected from each household to complete the individual questionnaire. The Individual Questionnaire contained questions about individual characteristics; smokeless tobacco use; smoking tobacco and cessation; secondhand smoke; tobacco-economics; media; and knowledge, attitudes, and perceptions about tobacco. The detailed methodology for GATS 1 and GATS 2 are published elsewhere [19, 20].

### Data collection

For this study, data was obtained from the website of Centers for Disease Control and Prevention, Atlanta. The data is open access available under the name of Global Tobacco Surveillance System DATA (GTSS Data) which was downloaded after registering with the website.

### Study participants

Adult females aged 15 years and above from the GATS 1 and GATS 2 formed the participants for this study. Available data of all females from both the rounds of GATS were analyzed for this study.

### Sample size

In GATS 1, 35,529 female participants were surveyed whereas a total of 40,265 females participated in the GATS 2 which formed the sample for this study.

### Outcome variable

The main outcome variable for analysis was smokeless tobacco use. Smokeless tobacco users (SLT Users) and smokeless tobacco non users (SLT Non users) have been classified based on the question “Do you currently use smokeless tobacco on a daily basis, less than daily, or not at all?”. All the participants who answered SLT use to be ‘daily’ or ‘less than daily’ were classified under SLT users and participants who replied as ‘not at all’ were listed as SLT Non users.

### Independent variables

In this study, relationship of smokeless tobacco was assessed with the following independent variables: age, residential area (urban/rural), regions of the country (North, Central, East, North-East and West), educational qualification, occupation, wealth index and smoking tobacco (Yes/No). Age was grouped into four categories 15 to 30 years, 31 to 45 years, 46 to 60 years and more than 60 years. Education was stratified into five categories as those were illiterate (no formal education); incomplete primary school education; completed primary education but not secondary school formed by incorporating “primary school completed” and “less than secondary school completed” from original data; secondary and higher secondary formed by secondary and higher secondary school completed; and graduation and above by merging “college/university completed” and “post graduate degree completed”. Further, occupation was classified into six group, the first being government and non-government employees formed by merging the government employees and non-government employees as per the original data followed by Daily wage/ casual laborer. Separate data was not available for daily wage/ casual laborer group in the GATS 1 survey hence, daily wage/ casual laborer group was included only for GATS 2 analysis in this study. The further categories under occupation formed were self-employed, student, homemaker and unemployed which was formed by clubbing retired, unemployed but able to work and unemployed but unable to work.

All participants smoking daily or less than daily were grouped as smokers whereas participants who do not smoke at all were clubbed as non-smokers for the analysis. Wealth index was analyzed using Principal Component Analysis based on the possession of household assets through GATS India data. Based on the scores of component analysis, all households were divided into five quintiles. Further, quintile with the highest scores were categorized as rich class, quintile with minimum scores as poor class and the middle three strata as middle class. We took middle class to be more as they represent in the population.

Further, we also analyzed regional variations in the number of women who are willing to quit and attempted to quit SLT. For “willing to quit”, we combined all participants who responded that they want to quit within next month or within next 12 months or quit someday, but not within next 12 months and all those who responded that they were not willing to quit as “not willing to quit”. For attempted to quit, we categorized “During the past 12 months, have you tried to stop using smokeless tobacco?” with ‘yes’ as attempted and ‘no’ as not attempted.

### Statistical analysis

Data were analyzed using STATA version 16.0 (STATA Corp., Texas) and R software (version 4.1.0) for graphs. Mean and Standard Deviation represents the measures of central tendency and dispersion for age. Frequency and proportion (%) for types of SLT used, socio-demographic characteristics of the participants with their exposure to SLT, are reported. Chi-square test was used to measure the significance between prevalence of GATS-1 and GATS-2. Correlates of SLT exposure were assessed separately using binary logistic regression. The variables which had *p* < 0.1 were included in the multivariable logistic regression analysis. We analyzed independent risk factor for all covariates and considered each covariate in regression model. Further, GATS 1 & 2 data were analyzed separately to calculate and compare Odds Ratio (Strength of Association) for each covariate separately. The association is expressed as Adjusted Odds ratio with 95% confidence interval (CI). Sampling weights were considered during analysis for both descriptive and regression models. For all weighted proportions, we added CI as the measure of uncertainty.

### Ethical considerations

This study is based on secondary data obtained from GATS 1and GATS 2 and hence, there is no participant risk. The data used is properly acknowledged and referenced wherever required.

## Results

**Fig. 1:**
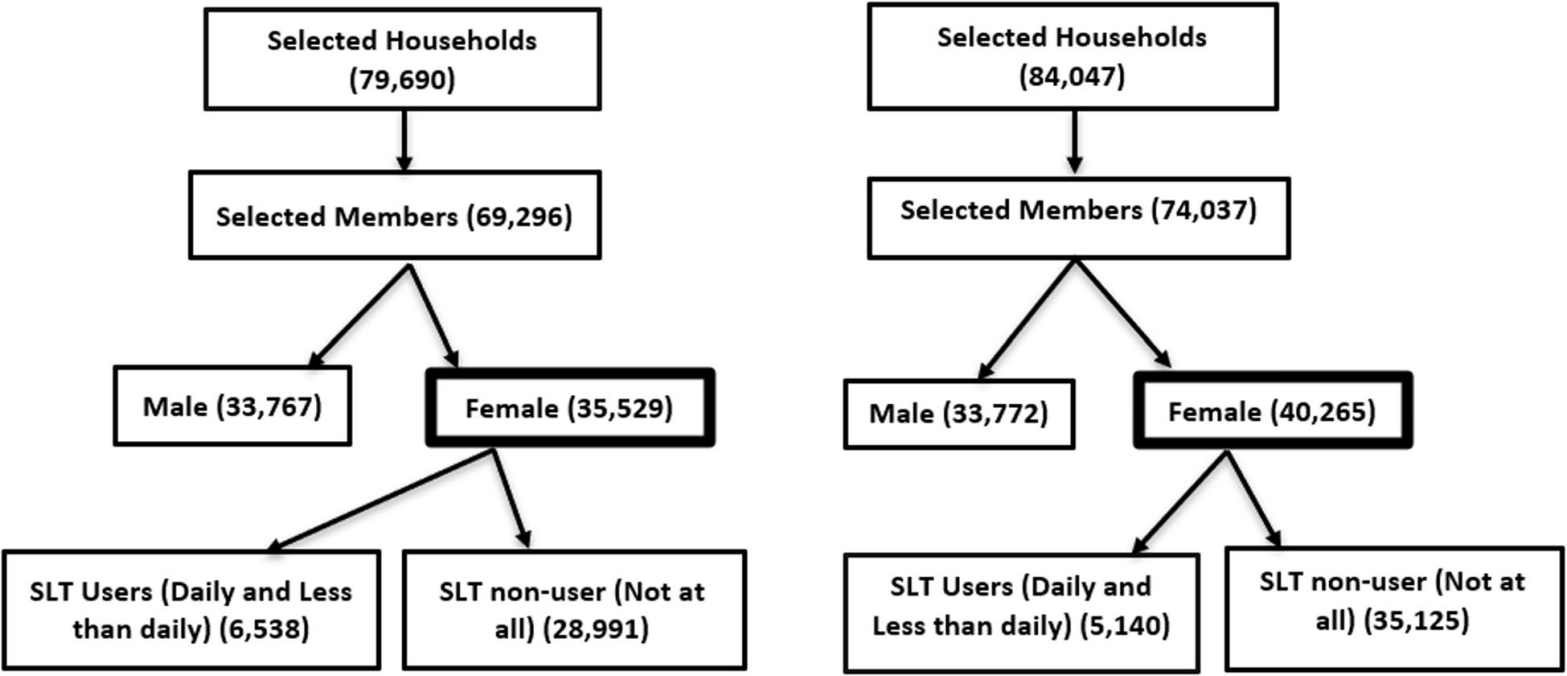
Selection criteria for 1a. GATS-1 and 1b. GATS-2.

There were 69,296 and 74,037 total participants (≥15 years) in GATS-1 and GATS-2 respectively. We included 35,529 and 40,265 female participants from both the surveys for analysis (Fig. 1). In the GATS-1, there were 14.91% daily SLT users and 3.49% less than daily SLT users, whereas in GATS-2, there were 11.09% daily SLT users and 1.68% less than daily SLT users making the prevalence of SLT use to be 18.4 and 12.8% respectively (Table 1). There was a significant change (*p*-value < 0.001) in SLT use among the women between GATS-1 and GATS-2. The mean age of female participants in GATS-1 was 36 (±16) years and that in GATS-2 it was 39 (±15.3) years. The mean initiation age of SLT among females was 22.7 (±12.2) years and 24.5 (±11.7) years for GATS-1 and GATS-2 respectively.

**Table 1:** Weighted prevalence of SLT users and non-users in GATS-1 and GATS-2

GATS 1 (*n* = 35,529)		GATS 2 (*n* = 40,265)		*p*- value
SLT Users n, % (CI)	SLT Non-Users n, % (CI)	SLT Users n, % (CI)	SLT Non-Users n, % (CI)	< 0.001
6538, 18.4 (18.0–18.8)	28,991, 81.6 (81.2–82.0)	5140, 12.8 (12.4–13.1)	35,125, 87.24 (86.9–87.6)

We observed oral tobacco products such as gul gudakhu (5.82%) to be most commonly used in GATS-1 whereas betel quid with tobacco product (3.91%) were more prevalent during GATS-2. The detailed prevalence of different types of SLT products are presented in Table 2. After sub-group analysis, we found that the majority of the women SLT users were > 60 years of age, belonged to rural areas and were minimally or not at all educated. Participants had varied job profiles and belonged to contrasting economic backgrounds.

**Table 2:** Weighted prevalence of different types of Smokeless Tobacco used

Types of SLT	GATS-1 (*N* = 35,529)	GATS-2 (*N* = 40,265)
	n,% (CI)	n,% (CI)
Betel quid with tobacco product	1541, 4.34 (4.13–4.55)	1575, 3.91 (3.72–4.11)
Khaini or tobacco lime mixture	1602, 4.51 (4.30–4.73)	1492, 3.71 (3.52–3.89)
Gutkha /tobacco lime, areca nut mixture	875, 2.46 (2.30–2.63)	888, 2.21 (2.06–2.35)
Oral tobacco, such as snuff and gul gudakhu	2069, 5.82 (5.58–6.07)	1560, 3.87 (3.69–4.07)
Pan masala and betel quid without tobacco	489, 1.38 (1.26–1.50)	374, 0.93 (0.84–1.03)
Nasal use of snuff	374, 1.05 (0.95–1.16)	206, 0.51 (0.44–0.59)
Others	963, 2.71 (2.54–2.88)	72, 0.18 (0.14–0.23)

Though, the overall prevalence has reduced, a surge was identified in North Eastern population where prevalence in SLT use increased from 29.2 to 35.0%. Among the northern population, SLT users increased by a minimal margin (1.3% during GATS-1 to 1.73% during GATS-2). Almost similar prevalence was observed among the self-employed females (23.6%), Government (Govt.) or Non-Govt. service class (25.1%) and unemployed (28.93%) during the first round of GATS. This situation improved with the course of GATS-2, but still a substantial portion of the unemployed cohort (22.56%) and self-employed (16.43%) were under the influence of smokeless tobacco. A new sub-group, daily wage/casual laborer, introduced during GATS-2 was the largest contributor (22.7%) of SLT use. Amongst tobacco smokers, a colossal part (36.7% in GATS-1) were found to be users of smokeless tobacco also (Table 3) representing the dual use of tobacco.

**Table 3:** Distribution of socio-demographic characteristics across SLT users in GATS-1 and GATS-2

Socio-demographic characteristics	Categories	GATS 1		GATS 2		Odds Ratio	
		Total n	SLT user n,% (CI)	Total n	SLT user n,% (CI)	GATS-1	GATS-2
Age in years	15–30	16,545	1770, 10.7 (10.2–11.2)	16,261	849 5.2 (4.9–5.6)	Ref.	Ref.
	31–45	10,312	2157, 20.9 (20.1–21.7)	12,255	1659 13.5 (12.9–14.2)	2.207 (1.984–2.455)	2.841 (2.490–3.242)
	46–60	5709	1619, 28.4 (27.2–29.5)	7197	1469 20.4 (19.5–21.4)	3.304 (2.928–3.728)	4.656 (4.077–5.317)
	> 60	2963	992 33.5 (31.8–35.2)	4552	1163 25.6 (24.3–26.8)	4.203 (3.629–4.868)	6.233 (5.344–7.270)
Residence	Urban	10,234	1140 11.1 (10.5–11.8)	13,773	1183 8.6 (8.1–9.1)	Ref.	Ref.
	Rural	25,295	5398 21.3 (20.8–21.9)	26,492	3957 14.9 (14.5–15.4))	2.163 (1.983–2.359)	1.869 (1.654–2.111)
Region	North	1752	23 1.3 (0.8–2.0)	3414	59 1.73 (1.3–2.2)	0.970 (0.745–0.126)	0.199 (0.151–0.261)
	Central	11,337	2118 18.7 (18.0–19.4)	11,627	1675 14.4 (13.8–15.1)	1.654 (1.447–1.891)	1.900 (1.637–2.207)
	East	7593	2171 28.60 (27.6–29.6)	8697	1295 14.9 (14.1–15.7)	2.884 (2.520–3.300)	1.976 (1.707–2.286)
	North-East	1294	378 29.2 (26.7–31.8)	1499	524 35.0 (32.5–37.4)	2.970 (2.611–3.377)	6.064 (5.248–7.007)
	West	5166	826 16.0 (15.0–17.0)	5971	850 14.2 (13.4–15.1)	1.371 (1.184–1.587)	1.875 (1.566–2.244)
	South	8387	1022 12.2 (11.5–12.9)	9057	737 8.1 (7.6–8.7)	Ref.	Ref.
Educational Qualification	No Formal Education	15,216	4376 28.8 (28.0–29.5)	14,725	3331 22.6 (22.0–23.3)	27.294 (19.367–38.467)	24.321 (13.082–45.218)
	Primary Incomplete	3824	846 22.1 (20.8–23.5)	3458	563 16.3 (15.1–17.6)	19.213 (13.436–27.474)	16.173 (8.591–30.446)
	Primary but not Secondary	8901	1006 11.3 (10.7–12.0)	10,079	957 9.5 (8.9–10.1)	8.614 (6.050–12.264)	8.724 (4.665–16.316)
	Secondary and Higher Secondary	5352	232 4.3 (3.8–4.9)	8316	241 2.9 (2.6–3.3)	3.060 (2.086–4.489)	2.480 (1.277–4.816)
	Graduation and Above	2143	31 1.4 (1.0–2.1)	3648	43 1.19 (0.9–1.6)	Ref.	Ref.
Occupation	Govt. and Non-Govt. employee	4867	1222 25.1 (23.9–26.3)	1882	166 8.8 (7.6–10.2)	7.561 (5.365–10.658)	11.450 (7.011–18.701)
	Daily wage/Casual labourer	NA	NA	5611	1272 22.7 (21.6–23.8)	NA	34.782 (22.581–53.576)
	Self-employed	4641	1094 23.6 (22.4–24.8)	2818	463 16.43 (15.1–17.9)	6.957 (4.926–9.825)	23.346 (14.931–36.503)
	Student	3184	135 4.2 (3.6–5.0)	3863	32 0.84 (0.7–1.2)	Ref.	Ref.
	Homemaker	21,543	3712 17.23 (16.7–17.7)	24,332	2815 11.6 (11.2–12.0)	4.696 (3.366–6.553)	15.531 (10.153–23.758)
	Unemployed	1202	348 28.93 (26.4–31.6)	1739	392 22.56 (20.6–24.6)	9.183 (6.223–13.550)	34.592 (21.739–55.044)
Wealth Index	Poor class	6643	524 7.9 (7.3–8.6)	9766	689 7.06 (6.55–7.58)	Ref.	Ref.
	Middle class	18,244	3426 18.8 (18.2–19.4)	23,223	2968 12.78 (12.35–13.22)	2.670 (2.340–3.114)	1.930 (1.669–2.231)
	Rich class	10,452	2567 24.6 (23.7–25.4)	6649	1349 20.29 (19.33–21.28)	3.801 (3.273–4.414)	3.352 (2.874–3.910)
Smoking Tobacco	Yes	1047	384 36.7 (33.8–39.7)	789	204 25.9 (22.8–29.1)	2.663 (2.153–3.294)	2.441 (1.885–3.160)
	No	34,482	6154 17.8 (17.4–18.3)	39,476	4936 12.5 (12.2–12.8)	Ref.	Ref.

Unadjusted univariate association of each of the covariates with SLT use was calculated as presented in the Table 3. Age is found to be directly associated with SLT use, whereas, educational qualification had an inverse relation. The odds ratio was 27.294 (19.367–38.467) and 24.321 (13.082–45.218) for those who never went to school in GATS-1 and 2 respectively.

Multivariable regression analysis (Table 4) showed the chances of SLT use increased among all age groups during GATS-2 survey with respect to the reference age group (15 to 30 years) than GATS-1. Chances of being a SLT user was highest among the North-Eastern women in both rounds [AOR: 4.567 (3.942–5.292) during GATS-1 and 9.149 (7.722–10.839) during GATS-2]. The odds of being a SLT user in rural areas, as compared to urban residents, had reduced from first round [AOR: 1.218 (1.103–1.347)] to the second round of GATS [1.022 (0.890–1.174)]. Adjusted Odds Ratio increased from GATS-1 to GATS-2 in Central [1.484 (1.287–1.711) to 2.244 (1.904–2.644)] and Western [1.567 (1.349–1.822) to 2.154 (1.789–2.594)] part of India, though only Eastern States of India showed a dip in AOR [3.006 (2.595–3.482) in GATS-1 vs. 2.242 (1.902–2.642) in GATS-2].

**Table 4:** Multivariable adjusted models for the association between SLT use and socio-demographic characteristics

Socio-demographic characteristics	Categories	AOR (95% CI)	
		GATS 1	GATS 2
Age	15–30 years	Ref.	Ref.
	31–45 years	1.667 (1.484–1.874)	1.898 (1.638–2.198)
	46–60 years	2.380 (2.077–2.726)	2.941 (2.536–3.411)
	> 60 years	3.112 (2.615–3.704)	3.691 (3.090–4.409)
Residence	Urban	Ref.	Ref.
	Rural	1.218 (1.103–1.347)	1.022 (0.890–1.174)
Region	North	0.105 (0.080–0.138)	0.267 (0.201–0.355)
	Central	1.484 (1.287–1.711)	2.244 (1.904–2.644)
	East	3.006 (2.595–3.482)	2.242 (1.902–2.642)
	North-East	4.567 (3.942–5.292)	9.149 (7.722–10.839)
	West	1.567 (1.349–1.822)	2.154 (1.789–2.594)
	South	Ref.	Ref.
Educational Qualification	No Formal Education	15.334 (10.783–21.806)	11.358 (5.984–21.558)
	Primary Incomplete	12.472 (8.675–17.931)	8.792 (4.593–16.829)
	Primary but not Secondary	7.153 (5.006–10.219)	6.499 (3.423–12.339)
	Secondary and Higher Secondary	3.273 (2.227–4.811)	2.700 (1.370–5.321)
	Graduation and Above	Ref.	Ref.
Occupation	Govt. and Non-Govt. employee	2.291 (1.584–3.314)	1.118 (0.801–1.562)
	Daily wage/Casual labourer	NA	1.469 (1.206–1.791)
	Self-employed	1.847 (1.277–2.671)	0.180 (0.112–0.290)
	Student	Ref.	Ref.
	Homemaker	1.349 (0.947–1.921)	0.724 (0.605–0.866)
	Unemployed	1.385 (0.900–2.130)	0.991 (0.750–1.309)
Wealth Index	Poor class	Ref.	Ref.
	Middle class	1.508 (1.293–1.760)	1.235 (1.055–1.447)
	Rich class	1.399 (1.177–1.663)	1.354 (1.129–1.624)
Smoking Tobacco Use	Yes	1.182 (0.915–1.526)	1.175 (0.861–1.605)
	No	Ref.	Ref.

Educational level evolved as an important contributing factor for SLT use. Women with no formal education had the highest prevalence (28.8 and 22.6% respectively during 2009–10 and 2016–17) as well as highest chance using SLT in both rounds [AOR: 15.334 (10.783–21.806) during GATS-1 and 11.358 (5.984–21.558) during GATS-2], though both prevalence and AOR, tend to decline in the second round of GATS. A considerable change in AOR was observed across all occupational groups, while daily wage/casual laborer added in GATS 2 had highest prevalence [1.469 (1.206–1.791)]. One of the noticeable findings of our study was, economically well-off women were more addicted to smokeless tobacco (prevalence 24.6 and 20.29% respectively in GATS-1 & GATS-2) as compared to the middle and least wealthy class, although the trend of SLT use reduced amongst all the groups. Dual use of tobacco i.e., use of smokeless tobacco with smoking tobacco was comparatively higher but it decreased over time (AOR: 1.182 (0.915–1.526) in GATS-1 and 1.175 (0.861–1.605) in GATS-2) though the changes are not so catchy.

**Fig. 2:**
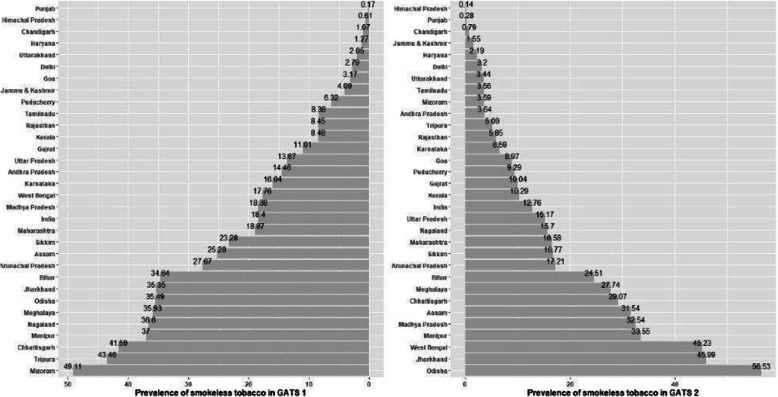
State level change in prevalence of smokeless tobacco across GATS 1 & 2

A state level analysis of prevalence of SLT use among women revealed Odisha had highest prevalence of 56.53% while Himachal Pradesh had lowest 0.14% during the recent GATS 2 survey (Fig. 2). Punjab, Himachal Pradesh ad Chandigarh continued to have least prevalence of SLT in both the rounds while the prevalence has considerably increased in states like Jharkhand, Odisha and West Bengal during GATS 2. Further, the regional differences (Table 5) across participants willing to quit and attempted to quit revealed a high percentage 33.3% vs. 34.80% were willing to quit tobacco whereas 41.48% vs. 41.7% of the participants attempted to quit in Central region across both rounds of survey. But, this trend inversed in Southern and Eastern parts of country.

**Table 5:** Regional differences among willing to quit and attempted to quit tobacco

Region	GATS 1		GATS 2	
	Willing to quit (*n* = 2399)	Attempted to quit (*n* = 1738)	Willing to quit (*n* = 2331)	Attempted to quit (*n* = 1500)
	n,% (CI)	n,% (CI)	n,% (CI)	n,% (CI)
North	5, 0.2 (0.07–0.48)	5, 0.29 (0.09–0.67)	41, 1.76 (1.27–2.38)	28, 1.87 (1.24–2.69)
Central	799, 33.3 (31.42–35.23)	721, 41.48 (39.15–43.84)	811, 34.80 (32.86–36.77)	626, 41.7 (39.22–44.28)
East	902, 37.62 (35.66–39.57)	281, 16.17 (14.47–17.98)	483, 20.74 (19.09–22.42)	325, 21.66 (19.61–23.84)
North-East	118, 4.91 (4.09–5.86)	73, 4.20 (3.30–5.25)	327, 14.03 (12.64–15.50)	124, 8.30 (6.92–9.78)
West	228, 9.5 (8.36–1.07)	320, 18.41 (16.62–20.32)	363, 15.58 (14.12–17.11)	188, 12.52 (10.90–14.32)
South	347, 14.46 (13.08–15.94)	338, 19.45 (17.61–21.39)	306, 13.11 (11.78–14.57)	209, 13.95 (12.22–15.79)

## Discussion

This study provided an overview of smokeless tobacco prevalence and its trend among adult women in India using a nationally representative data from two rounds of GATS Survey. The mean initiation age of SLT among females was 22.7 (±12.2) years and 24.5 (±11.7) years for GATS-1 and GATS-2 respectively which is consistent with the findings of a study which reports mean initiation age for any SLT product to be 20.1 (±7.5) years among women [21]. This also indicated that females tend to initiate SLT use late than their male counterparts whose mean initiation age is reported around 17.24 (±2.18) years [22]. This study reported an overall relative reduction in prevalence of SLT use among adult women in India by about 5% between GATS 1 and GATS 2 which signifies a large number of people want to quit. Further, prevalence of SLT use among older adults aged > 60 years appeared to be more than the younger generation which is a positive indicator as fewer young people tend to be habitual of tobacco. A substantial part of rural women indulging in SLT use is of great concern. Although, comparing GATS 1 and GATS 2, we assessed percentage reduction in SLT use among rural women was more as compared to urban women indicating their willingness to quit. Hence, more culturally acceptable and linguistically focused programs need to be designed to provide them assistance in quitting.

Tobacco usage varies across different regions of India where North-east India majorly contributes in SLT burden followed by East and Central India. This can be attributed to varied levels of socio-economic development throughout India However, Eastern India has successfully managed to maximally reduce the burden to almost half. This also provides an opportunity to systematically study the factors behind this change and replicate the strategy in other regions too. Education is an important correlate for SLT use in India as this study reported a direct relation between SLT use and level of education. Women with no formal education had highest prevalence of SLT use. This can be attributed to lack of knowledge and various perceptions about the use of SLT in community. Daily wage/casual laborers and unemployed women appear to consume SLT more. Several studies suggest people tend to use tobacco either in free time or when they need to work hard to get a sense of mental relaxation [23]. Here, a noticeable increase in SLT users among the rich strata is observed which strengthens the notion that women being socially and economically more independent have recently become the soft target for tobacco industry. This clearly emphasizes gender based tobacco control policies [24] are required which will not only target for health promotion but also empower women to take informed decisions for their health which in turn affects their family’s health too.

Dual use of tobacco both as SLT and smoking is widely believed to go hand in hand which is supported by this study too [25]. Here, we observed the prevalence of SLT use (36.7% & 25.9%) increased concomitantly among the smokers. Dual use of tobacco is potentially one of the highest risk factors for ill effects on general and reproductive health among women. It is worth noting here that 4.91% vs. 14.03% were willing to quit tobacco and 4.20% vs. 8.30% attempted to quit in North Eastern region across both rounds of survey. This signifies, despite a noticeable surge in prevalence of SLT, the number of people willing to quit and attempted to quit have almost doubled during GTAS 2. This can be treated as an opportunity for policy makers to take lead and help people in quitting. Also, the number of SLT users have considerably increased in states such as Jharkhand, Odisha and West Bengal during GATS 2 which needs a fair attention.

Various tobacco control programmes and policies such as National Tobacco Control Programme (NTCP), 2007–08 and The Cigarettes and Other Tobacco Products (Prohibition of Advertisement and Regulation of Trade and Commerce, Production, Supply and Distribution) Act, 2003 (COTPA) are in implementation in India. India is also a party to the World Health Organization Framework Convention on Tobacco Control (WHO FCTC). NTCP targets to create awareness related to ill effects of tobacco, reduce production and supply of tobacco products and ensure effective implementation of COTPA. It also aims to help people quit tobacco through the WHO FCTC framework. Although, these provisions have helped in reducing the tobacco use but they do not focus on gender dimensions. Hence, based on this study we recommend following tobacco control measure for this group.

### Recommendations

Gender-based tobacco control policies with bottom-up approach with participation of women should be framed in order to make it culturally and socially acceptable. More tobacco research with greater representation of women is required to generate substantial evidence in this field. Tobacco control programs may aim at women evenly and help them in cessation. Socio-behavioral and cultural factors related to females need to be addressed while framing any regulations for tobacco control. Community based awareness and prevention should aim to reach both males and females equally. Awareness materials should be developed in local language along with more illustrations in order to reach all strata of society. Health professionals should screen and advice for tobacco cessation to all patients attending healthcare centers so that this opportunity is not missed.

### Strengths and Limitations

As per our knowledge, this is the first study to demonstrate trends of smokeless tobacco use among Indian women. We compared the data from GATS-1 & GATS-2, a nationally representative exclusive adult tobacco survey containing all possible correlates such as age, sex, region, occupation etc. During our study, we were unable to take into account some additional factors like alcoholism, stress, or other habitual and behavioral practices due to data deficit.

## Conclusions

Tobacco control measures have helped in reducing SLT use among women in India over the years but this change needs further pace. Hence, more focused gender-based tobacco control programs and policies should be advocated so that women, a neglected group in tobacco control, do not fall prey in the hands of legalized tobacco industry. More focused gender-based tobacco research to generate concrete evidence is requirement of the time.

## Data Availability

The datasets generated during and/or analyzed during the current study are available in the Global Tobacco Surveillance System Data (GTSSData) repository, (https://nccd.cdc.gov/GTSSDataSurveyResources/Ancillary/DataReports.aspx?CAID=2).

## Abbreviations

SLT: Smokeless Tobacco
NFHS: National Family Health Survey
NSS: National Sample Survey
NHSDAA: National Household Survey of Drug and Alcohol Abuse in India
GATS: Global Adult Tobacco Survey
PSU: Primary Sampling Unit
PPS: Probability Proportional to Size
CEB: Census Enumeration Blocks
GTSS Data: Global Tobacco Surveillance System DATA
